# Electronic hybridisation implications for the damage-tolerance of thin film metallic glasses

**DOI:** 10.1038/srep36556

**Published:** 2016-11-07

**Authors:** Volker Schnabel, B. Nagamani Jaya, Mathias Köhler, Denis Music, Christoph Kirchlechner, Gerhard Dehm, Dierk Raabe, Jochen M. Schneider

**Affiliations:** 1 Materials Chemistry, RWTH Aachen University, Kopernikusstr 10, D-52074 Aachen, Germany; 2 Max-Planck-Institut für Eisenforschung, Max-Planck-Straße 1, D-40237 Düsseldorf, Germany

## Abstract

A paramount challenge in materials science is to design damage-tolerant glasses. Poisson’s ratio is commonly used as a criterion to gauge the brittle-ductile transition in glasses. However, our data, as well as results in the literature, are in conflict with the concept of Poisson’s ratio serving as a universal parameter for fracture energy. Here, we identify the electronic structure fingerprint associated with damage tolerance in thin film metallic glasses. Our correlative theoretical and experimental data reveal that the fraction of bonds stemming from hybridised states compared to the overall bonding can be associated with damage tolerance in thin film metallic glasses.

The combination of high strength and toughness, which constitutes damage tolerance, appears to be self-excluding; tough materials need to dissipate energy, for example, by plasticity, whereas high-strength materials are designed to prevent plastic deformation^1^. Hence, materials exhibiting concomitant high toughness (high resistance to bond breakage by shear deformation) and high strength, or more precisely, high yield stress (high resistance for shear deformation), are chimeric from the perspective of chemical bonding. Demetriou *et al*. reported on a damage-tolerant metallic glass that combines strength and toughness beyond the benchmark ranges established by any known material^2^; however, its underlying physical and chemical mechanisms are not understood on the atomic scale. Schroers and Johnson^3^ suggested that a large Poisson ratio and a low glass transition temperature might be indicators of ductile behaviour in bulk metallic glasses. It was proposed that a large Poisson’s ratio allows for a shear collapse before the extensional instability of crack formation can occur^3^. Current alloy design guidelines for metallic glasses are based on the notion of a universal relationship between the Poisson ratio and fracture energy to define the brittle-ductile transition^4^,^5^. However, Poisson’s ratio by definition describes only elastic behaviour; for materials with a small Poisson’s ratio and hence a small bulk-to-shear modulus ratio, a pressure-induced elastic volume change is favoured over a shear-induced shape change. On the other hand, materials exhibiting a large Poisson’s ratio resist volume change in favour of shear-induced shape change. Hence, there is an on-going discussion on the reliability of using the Poisson ratio to determine fracture toughness values, as presented in the literature^6^,^7^,^8^,^9^,^10^,^11^. Raghavan *et al*.^11^ have reported that a Poisson ratio-toughness correlation does not hold. A phenomenological criterion based on a critical fictive temperature was proposed by Kumar *et al*.^8^. They were able to predict room-temperature mechanical behaviour and its sensitivity to the cooling rate and annealing-induced embrittlement for bulk metallic glasses^8^. For the Ti-Zr-Cu-Pd system, it has been reported that by alloying with In, the plastic strain can be increased up to 10.2%, which is proposed to be caused by increased atomic-scale structural heterogeneity^10^. It is inferred by Zheng *et al*.^10^ that substitution with relatively large atoms renders the bonding nature more metallic and less shear-resistant.

To identify the electronic fingerprint associated with the toughness of thin film metallic glasses, we have critically appraised the design proposal of Lewandowski *et al*.^5^ and Greaves *et al*.^4^ using a correlative *ab initio* molecular dynamics and an *in situ* micro mechanical testing approach while also considering the data in the literature. Furthermore, we have included all available literature data that contains information on the mechanical properties and electronic structure in the discussion^5^,^12^,^13^,^14^,^15^,^16^,^17^. We have synthesised Pd_57.0_Al_23.9_Cu_11.4_Y_7.7_, Co_42.0_Fe_13.2_Zr_10.7_Ta_6.6_B_27.5_, Co_58.4_Fe_7.0_B_34.6_, Cu_67.8_Zr_32.2_ and Co_68.3_Zr_31.7_ metallic glass thin films, which cover the brittle to ductile Poisson’s ratio transition range proposed in the literature^5^. To represent a broad variety of bonding types, the selection of metallic glasses includes both metalloid and non-metalloid metallic glasses. For the systems Pt_57.5_Cu_14.7_Ni_5.3_P_22.5_^3^ and Au_49_Ag_5.5_Pd_2.3_Cu_26.9_Si_16.3_^18^ synthesised and tested in the literature, we have complemented the reported mechanical properties with information on the electronic structure. While several authors^11^,^18^,^19^ report data in conflict with Lewandowski *et al*.^5^ and Greaves *et al*.^4^, a comprehensive and general concept on the electronic structure level is lacking. However, the significance of the electronic structure for plastic behaviour was reported by He *et al*.^12^ and Yang *et al*.^13^, who stated that alloying with transition metals, which cause “stronger orbital hybridisation with Al”^12^, should be avoided for Zr-based glasses, whereas “clusters linked with metal-metal bonds”^13^ accommodate shear bands for Fe-based glasses^13^. The conclusions of the authors were therefore specific to the material system being considered, so no discussion regarding general relevance was undertaken in the literature^12^,^13^. It is the goal of this work to identify the electronic structure fingerprint associated with damage tolerance in thin film metallic glasses.

## Results

### Investigation of chemical homogeneity

**Figure 1:**
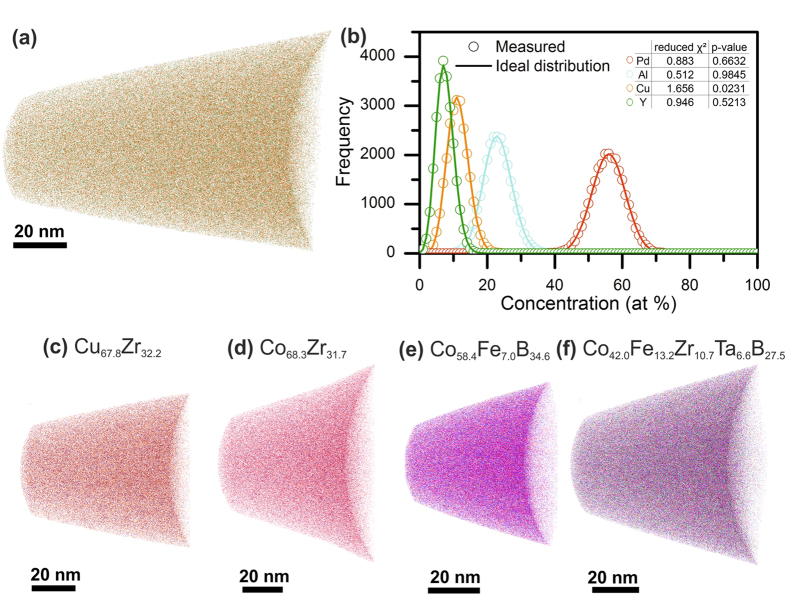
The spatially resolved chemical analysis by atom probe tomography reveals a homogeneous chemical distribution throughout the specimen. (**a**) The results for the Pd_57.0_Al_23.9_Cu_11.4_Y_7.7_ thin film metallic glass are shown. (**b**) The random distributions of all elements for the present concentration are displayed as lines; the measured values, which are in very good agreement with the random distributions, are represented by the open symbols. The inset table shows the deviation between the random and measured distributions; the p-value indicates its randomness^22^. (**c–f**) Tomography analysis of the Cu_67.8_Zr_32.2_, Co_68.3_Zr_31.7_, Co_58.4_Fe_7.0_B_34.6_ and Co_42.0_Fe_13.2_Zr_10.7_Ta_6.6_B_27.5_ thin film metallic glasses, respectively.

Park *et al*.^20^ and Zhao *et al*.^9^ showed that chemical inhomogeneity may cause embrittlement of metallic glasses and alter their mechanical response. Hence, we have chosen physical vapour deposition with a quenching rate^21^ of 10^15^ to 10^16^ K/s as the synthesis technique to avoid the formation of compositionally inhomogeneous regions caused e.g. by diffusion. We have probed the spatial elemental distribution of all thin film metallic glasses evaluated here by atom probe tomography. Figure 1a,b shows the tomography and frequency distribution analysis^20^ of the Pd_57.0_Al_23.9_Cu_11.4_Y_7.7_ glass^22^. Figure 1c–f shows the tomography analysis for the Cu_67.8_Zr_32.2_, Co_68.3_Zr_31.7_, Co_58.4_Fe_7.0_B_34.6_ and Co_42.0_Fe_13.2_Zr_10.7_Ta_6.6_B_27.5_ thin film metallic glass, respectively. It is evident that all alloying constituents are randomly distributed, and hence no strain localisation is expected. Spinodal decomposition-induced columnar growth was observed in the literature for combinatorially grown thin film metallic glasses by both atom probe tomography and high energy X-ray diffraction^23^. Here, no spinodal decomposition-induced columnar growth was observed by atom probe tomography for any of the film metallic glass investigations, as is observed in Fig. 1. The random distributions of all the elements may be due to the large quenching rates typical of physical vapour-deposited thin film metallic glasses^21^, eliminating the possible influence of chemical inhomogeneity on the mechanical properties of the thin film metallic glasses investigated here compared to the literature data for bulk metallic glasses^9^,^20^.

### *In situ* micro-cantilever deflection experiments

**Figure 2:**
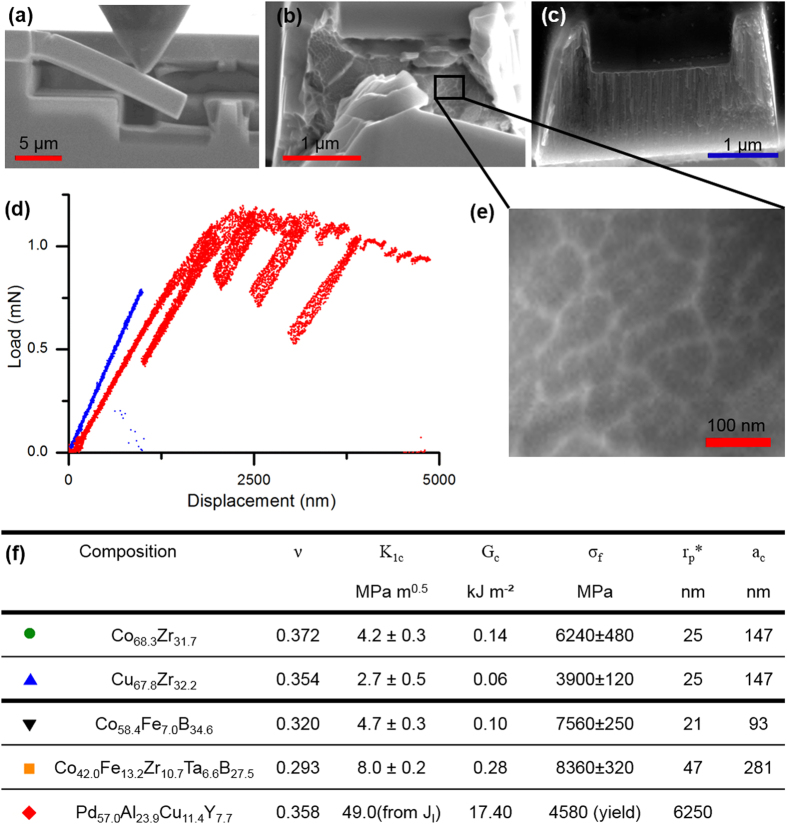
*In situ* micro-cantilever deflection experiments. The fracture strength and toughness of the metallic glass thin films are measured by employing both un-notched and pre-notched specimens. (**a**) Representative scanning electron image of an *in situ* micro-cantilever deflection experiment performed on a Pd_57.0_Al_23.9_Cu_11.4_Y_7.7_ glass. (**b**,**c**) Show high magnification scanning electron microscope images of the crack trajectory and fracture surface of the tough Pd_57.0_Al_23.9_Cu_11.4_Y_7.7_ and the brittle Cu_67.8_Zr_32.2_ metallic glass, respectively. (**d**) Load-displacement graphs for the brittle Cu_67.8_Zr_32.2_ in blue and the tough Pd_57.0_Al_23.9_Cu_11.4_Y_7.7_ in red. The dissipated energy before fracture (the area under the curve) for the highly damage-tolerant Pd_57.0_Al_23.9_Cu_11.4_Y_7.7_ glass is much higher compared to the brittle Cu_67.8_Zr_32.2_ glass. (**e**) Magnification of fracture surface morphology of the ductile Pd_57.0_Al_23.9_Cu_11.4_Y_7.7_ metallic glass thin film. (**f**) Summary of the *ab initio* calculated Poisson’s ratio (ν), and the measured quantities: fracture toughness (K_1c_), fracture energy (G_c_), fracture strength (σ_f_), plastic zone size (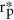) and critical defect size (a_c_) of the thin film metallic glasses studied within this work. The Pd-based glass yielded before fracture, hence the critical defect size could not be calculated for that case.

The mechanical behaviours of the thin film metallic glasses are examined by micro-fracture experiments^24^,^25^, as presented in Fig. 2. The metallic glass thin films exhibit a Poisson ratio range from 0.29 to 0.37. The load-displacement curves from the notched cantilevers for two representative examples of marginally damage-tolerant Cu_67.8_Zr_32.2_ and highly damage-tolerant Pd_57.0_Al_23.9_Cu_11.4_Y_7.7_ glass with the same Poisson ratio are depicted in Fig. 2d in blue and red, respectively. The load-displacement curves are not normalised against the cantilever cross-section. However, a clear difference in the nature of the fracture can be observed. The marginally damage-tolerant glass shows pure elastic deformation before an abrupt, catastrophic fracture at a peak load from which the fracture toughness (2.7 MPam^1/2^) and fracture energy (0.06 kJ/m^2^) were determined using linear elastic fracture mechanics^24^. The low damage tolerance of the Cu_67.8_Zr_32.2_ metallic glass thin film is in agreement with the qualitative brittle behaviour reported for bulk Cu_66_Zr_34_ and Cu_64_Zr_36_ metallic glasses^26^.

In contrast, the more damage-tolerant Pd_57.0_Al_23.9_Cu_11.4_Y_7.7_ glass exhibits extensive plastic deformation in the form of shear bands, leading to a significant energy dissipation before fracture^1^. Therefore, elasto-plastic fracture mechanics via J-integral analysis^25^ was applied to quantify the damage tolerance. The decrease in load upon continuous deformation for the ductile Pd_57.0_Al_23.9_Cu_11.4_Y_7.7_ thin film metallic glass may be explained by an increase in the crack depth, which is also indicated by the increase in compliance at every unloading step with increasing deformation. In addition, an intrinsic strain softening that is typical for ductile metallic glasses may occur, which would also be consistent with literature^27^,^28^. The determined fracture toughness (49 MPam^1/2^) and fracture energy (17.4 kJ/m^2^) significantly exceed the values for the brittle Cu_67.8_Zr_32.2_ glass. The fracture toughness measured for the Pd_57.0_Al_23.9_Cu_11.4_Y_7.7_ thin film metallic glass is 49 MPam^1/2^, which agrees well with reported literature values^5^ ranging from 29 to 67 MPam^1/2^ for Pd-based bulk metallic glasses. Fracture toughness values of 5.5 MPam^1/2^ are reported for bulk Co-based metallic glasses^29^, which are also in good agreement with the values of 4.2, 4.7 and 8.0 MPam^1/2^ reported for the Co_68.3_Zr_31.7_, Co_58.4_Fe_7.0_B_34.6_ and Co_42.0_Fe_13.2_Zr_10.7_Ta_6.6_B_27.5_ thin film metallic glasses investigated here.

A snapshot from the *in situ* scanning electron microscopy bending experiment on the Pd_57.0_Al_23.9_Cu_11.4_Y_7.7_ thin film metallic glass is shown in Fig. 2a. The fracture surfaces of the two glasses are shown in Fig. 2b,c and conform to the known topographies of ductile (large dimples) and brittle (fine features) metallic glass, respectively. We have used micro-cantilever bend tests to measure the fracture strengths of all the metallic glass thin films studied. Bending results in a strain gradient, and the volume that experiences the maximum tensile stress is very small and is close to the fixed end of the cantilever, which could be responsible for the high fracture strengths recorded compared to uniaxial tensile loading where a large volume is exposed to tensile strains. We calculated the critical defect size *a*_*c*_ expected in our samples using equation (1), which ranges between 93 nm for the Co_58.4_Fe_7.0_B_34.6_ and 281 nm for the Co_42.0_Fe_13.2_Zr_10.7_Ta_6.6_B_27.5_ metallic glass thin film:


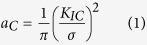


From the evaluation of the critical defect size, we can infer that for high-purity metallic glass thin films deposited by magnetron sputtering as in the present case, structural heterogeneities that are larger than 300 nm can be excluded. It is inferred that the small critical defect size combined with the probability of finding a surface defect in the limited volume that experiences maximum tensile stresses may be the cause of the high fracture strength observed. It has been shown that the fracture toughness for brittle materials can be evaluated down to the micron-scale^30^,^31^, which has routinely been reported for bulk Si^30^, thin film silicon-oxides, thin film silicon-nitrides, thin film silicon-oxynitrides and hard coatings^24^,^32^. Figure 2e shows a close-up of the fracture surface morphology of the ductile Pd_57.0_Al_23.9_Cu_11.4_Y_7.7_ metallic glass thin film. The largest feature size found on the fracture surface, which is another sign of heterogeneity, is 60 nm for the ductile Pd_57.0_Al_23.9_Cu_11.4_Y_7.7_ metallic glass thin film investigated. Hence, we infer that heterogeneities larger than 60 nm are not present. From the atom probe tomography analysis we can exclude chemically induced structural heterogeneities that are smaller than 60 nm^33^. Furthermore, density fluctuations, which can be a fingerprint for structural heterogeneities, are not observed by atom probe tomography^34^. Hence, structural heterogeneities that exhibit density fluctuations are not present for sizes larger than 2 nm. A variation in the local atomic packing causes structural heterogeneities in the sub-nm range^35^, which may lead to an increase in the number of shear transformation sites and hence an increase in ductility^35^,^36^,^37^. Owing to the fast quenching rates associated with physical vapour deposition^21^, we infer that the thin film metallic glasses synthesised here are all in similar states of low structural relaxation^8^. Hence, a tendency towards variations in the local atomic packing density is expected, which should result in an increase in the shear transformation zones^35^. However, only the Pd_57.0_Al_23.9_Cu_11.4_Y_7.7_ thin film metallic glass is observed to behave ductile. Figure 2f summaries the *ab initio* calculated Poisson’s ratio (ν), and the measured quantities, the fracture toughness (K_1c_), fracture energy (G_c_), fracture strength (σ_f_), plastic zone size (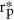), as defined by equation (2), and critical defect size (a_c_) of the glasses studied within this work. For all glasses except for the ductile Pd_57.0_Al_23.9_Cu_11.4_Y_7.7_ thin film metallic glass, the fracture strengths were used as lower limits of the yield strength to calculate the plastic zone size:


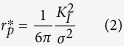


The focus ion beam (FIB)-machined notches were bridged to create real cracks upon reaching the fracture load. This type of notch was first introduced by Matoy *et al*.^24^ to circumvent the problem associated with a finite root radii on the fracture toughness measurements. By comparing the K_IC_ measured from the notch tip and crack tip using a clamped beam stable geometry, Jaya *et al*.^30^ were able to prove that FIB notches machined at low currents have a tip radius of less than 50 nm and behave as real cracks. Furthermore, Jaya *et al*. showed that the K_IC_ determined at the micron-scale in a brittle material such as Si using different test geometries is not different from its reported bulk values^30^. Hence, the sample size does not play a role as long as the size of the plastic zone is much smaller (at least 1/10th) than the cantilever dimensions^30^,^31^. Both the continuum-based plastic zone size calculations using equation (2) and the critical defect size measured from the fracture surface of the metallic glass thin films prove that our results are well within this range for the plane strain fracture toughness except for the results for the ductile Pd_57.0_Al_23.9_Cu_11.4_Y_7.7_ thin film metallic glass.

**Figure 3:**
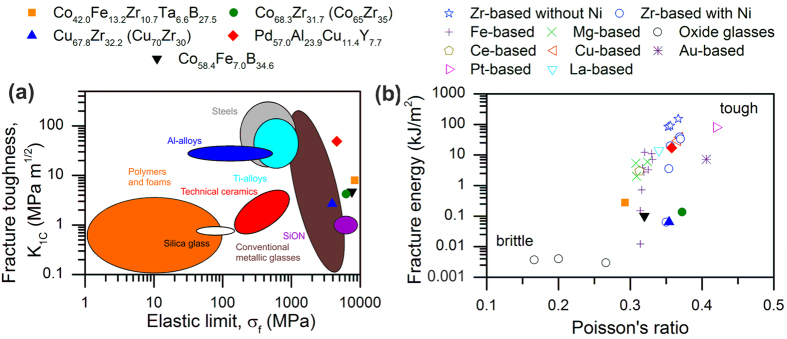
Experimental validation reveals extraordinary damage tolerance for the metallic glasses studied in this work. (**a**) The fracture strength of the thin film metallic glasses studied in this work is close to the ideal strength of the material. The Pd_57.0_Al_23.9_Cu_11.4_Y_7.7_ thin film metallic glass is shown to exhibit high damage tolerance. (**b**) The Poisson ratio cannot gauge the chemical origin of the rapid evolution from brittle to tough behaviour. Literature data for the Mg-based, Ce-based and La-based^18^,^42^, Fe-based^40^, oxide^5^,^43^, Zr-based^11^,^12^, Cu-based^6^,^41^, Pt-based^3^, Au-based^18^ and Ge-Se chalcogenide glasses^16^ are presented.

**Figure 4:**
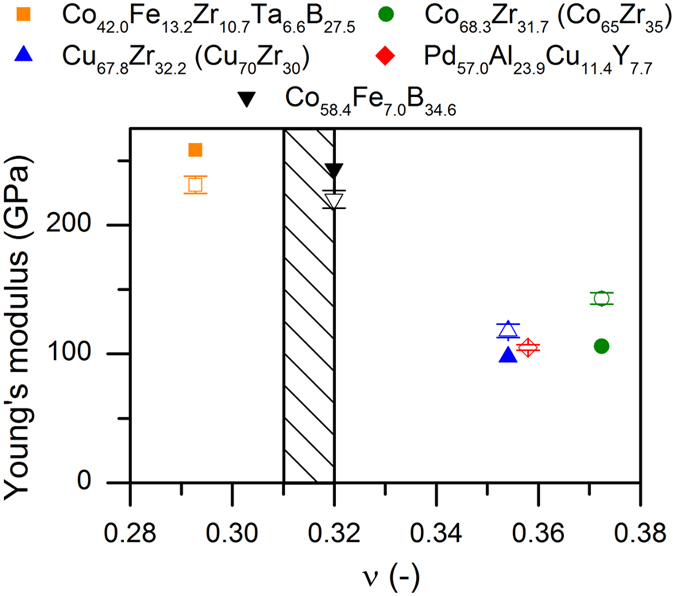
Measured and calculated Young’s modulus. The Young’s moduli obtained from the simulated and synthesised thin film metallic glasses are denoted by full and open symbols, respectively. The transition line proposed by Lewandowski *et al*.^5^ is included. However, two of the three systems, which should exhibit tough behaviour, are observed to be brittle.

The outstanding damage tolerance of the thin film metallic glasses investigated here compared to other material classes^24^,^38^,^39^ is evident in Figs 2f and 3. The fracture energy is depicted as a function of the calculated Poisson’s ratio for the five thin film metallic glasses studied here and 36 other glasses from the literature^3^,^5^,^6^,^11^,^12^,^14^,^15^,^16^,^18^,^19^,^40^,^41^,^42^,^43^. A good agreement between the measured and the calculated Young’s modulus can be observed in Fig. 4, which validates the quantum mechanical model.

It can be observed in Fig. 3b that the oxide glasses^5^,^43^ with a Poisson ratio of 0.266 and lower exhibit low toughness and have a fracture energy below 0.01 kJ/m^2^. The Fe-based metallic glasses display a chemically induced systematic change in the Poisson ratio from 0.314 to 0.330^40^. The data for the last three thin film metallic glasses in Fig. 2e are consistent with the present alloying guidelines^4^,^5^, whereas the first two thin film metallic glasses in Fig. 2e exhibit Poisson’s ratios of 0.354 to 0.372 and brittle behaviour. These Poisson’s ratios are much higher than the proposed sharp brittle-to-tough transition range of 0.31–0.32^5^. The three orders of magnitude difference in fracture energy between the tough Pd_57.0_Al_23.9_Cu_11.4_Y_7.7_ and the brittle Cu_67.8_Zr_32.2_ thin film metallic glasses with identical Poisson’s ratios is in conflict with the universal relationship proposed between the Poisson ratio and fracture energy^4^,^5^. The Au_49_Ag_5.5_Pd_2.3_Cu_26.9_Si_16.3_ system presented in Fig. 3b exhibits a low fracture energy compared to its high Poisson’s ratio of 0.406, which cannot be explained by extrinsic factors^18^. Hence, the interplay between the alloy compositions, Poisson’s ratio and toughness of metallic glasses is not fully understood^18^. Furthermore, it can be observed in Fig. 3b that the Zr-based metallic glasses exhibit two separate distributions of toughness dependence within the same Poisson’s ratio range^11^,^12^. The Zr-based metallic glasses without Ni (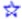)^12^ exhibit toughness values that exceed 140 kJ/m^2^, whereas the Zr-based metallic glasses containing Ni (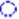)^11^,^12^ possess toughness values that range from 0.06 to 39 kJ/m^2^ (Fig. 3b). In addition, there are reports in the literature on Pd-based metallic glasses that exhibit a brittle-to-tough evolution within a narrow Poisson’s ratio range of 0.39–0.41^19^,^44^. Hence, it is evident from our data and literature data^6^,^7^,^8^,^9^,^10^ that the Poisson’s ratio range of 0.31 to 0.32 does not accurately gauge the brittle-to-ductile transition.

### Electronic structure of damage-tolerant thin film metallic glasses

**Figure 5:**
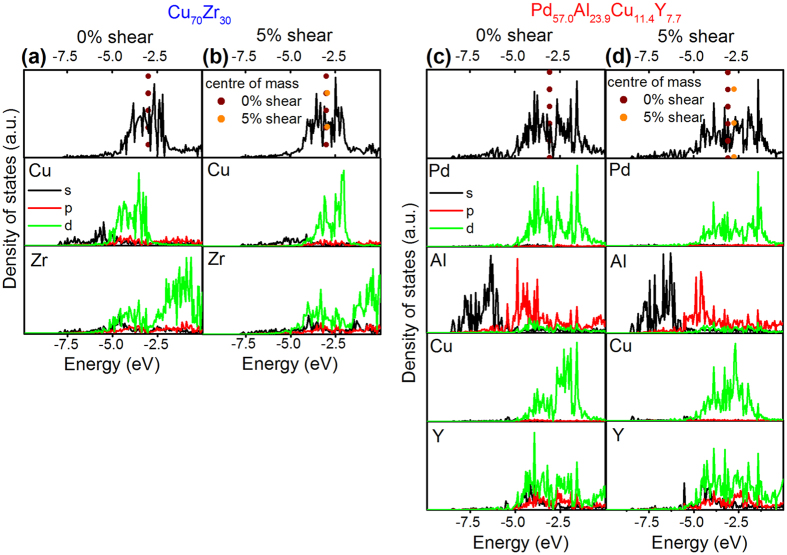
With a low degree of hybridisation, glasses are more likely to accommodate shear and facilitate the generation of shear transformation zones. (**a–d**) Density of states analysis for Cu_70_Zr_30_ and Pd_57.0_Al_23.9_Cu_11.4_Y_7.7_ in a sheared and un-sheared state. The s, p and d orbitals are presented in black, red and green, respectively. The Fermi level for each glass is set to zero. The energy levels down to −10 eV are presented. The vertical dotted lines indicate the shift of the centre of mass for the total density of states.

**Figure 6:**
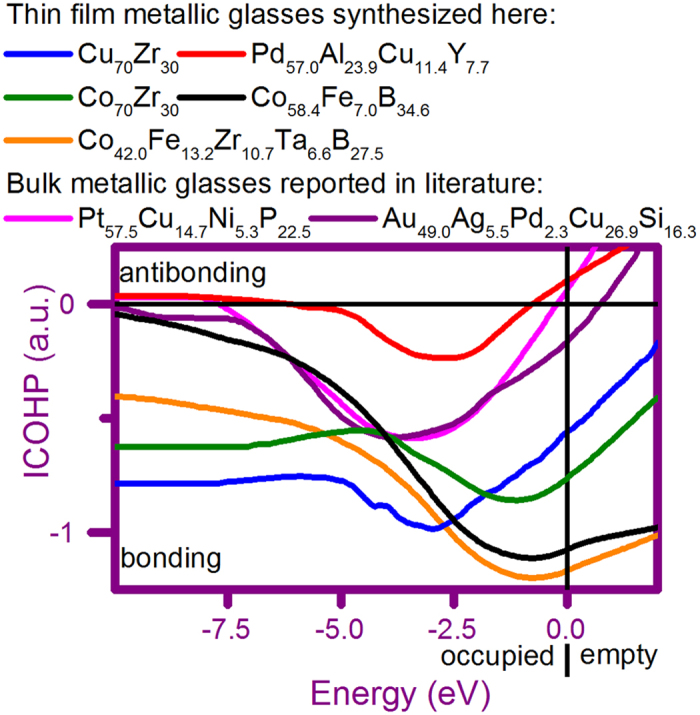
Bonding interactions for all systems evaluated. The density of states analysis is consistent with the bonding analysis in the form of the integrated crystal orbital Hamilton population (ICOHP). For the Pt_57.5_Cu_14.7_Ni_5.3_P_22.5_^3^ and Au_49_Ag_5.5_Pd_2.3_Cu_26.9_Si_16.3_^18^ bulk metallic glasses synthesised and tested in the literature, we have completed mechanical property reports along with electronic structure information.

**Figure 7:**
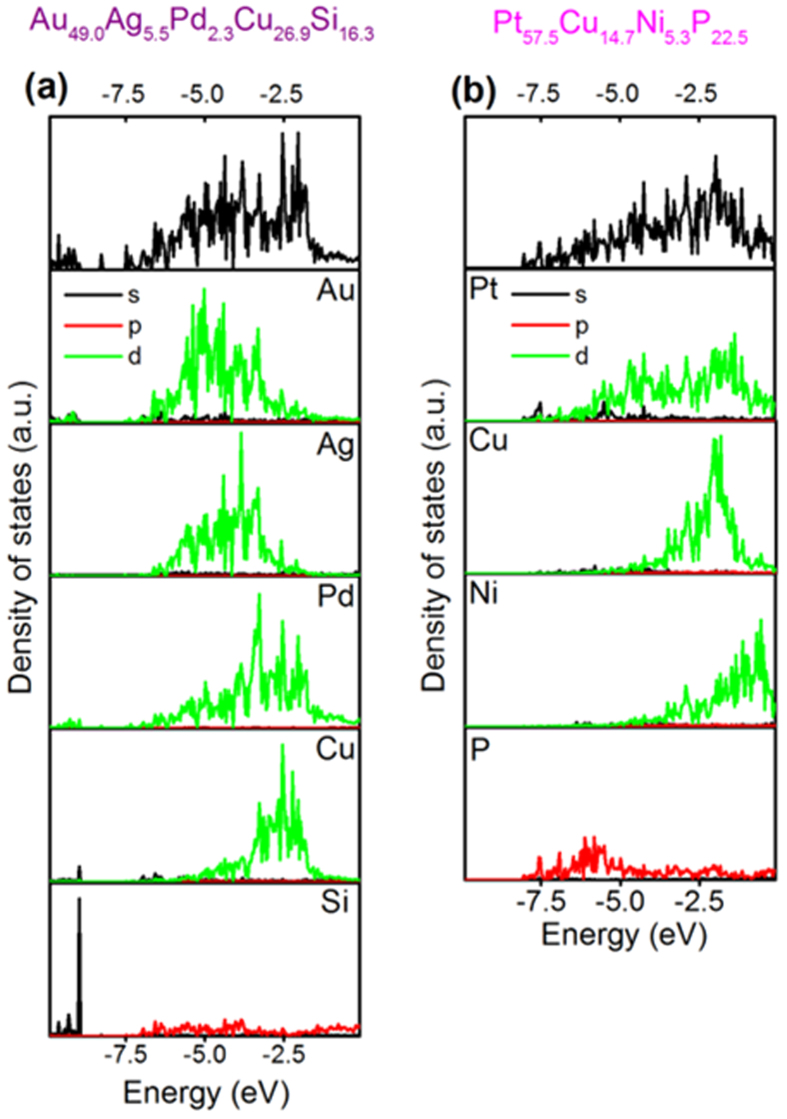
Total and partial density of states for the Au_49_Ag_5.5_Pd_2.3_Cu_26.9_Si_16.3_ and Pt_57.5_Cu_14.7_Ni_5.3_P_22.5_ bulk metallic glasses. (**a**) Density of states analysis for the Au_49_Ag_5.5_Pd_2.3_Cu_26.9_Si_16.3_ metallic glass, including partial density of states for Au, Ag, Pd, Cu and Si. (**b**) Density of states analysis for the Pt_57.5_Cu_14.7_Ni_5.3_P_22.5_ metallic glass, including partial density of states for Pt, Cu, Ni and P.

To investigate the electronic structure for the observed evolution of the toughness behaviour, we use density-of-state analysis to probe all five glass compositions. In addition, we have calculated the density-of-states for Au_49_Ag_5.5_Pd_2.3_Cu_26.9_Si_16.3_^18^ and Pt_57.5_Cu_14.7_Ni_5.3_P_22.5_^3^, which were reported to exhibit a low and a high fracture energy of 7.2 kJ/m^2^ and 61.3 kJ/m^2^, with Poisson’s ratios of 0.406 and 0.42, respectively. We present the results of two glass compositions that have identical Poisson’s ratios but exhibit the largest difference in fracture energies: the tough Pd_57.0_Al_23.9_Cu_11.4_Y_7.7_ and the brittle Cu_67.8_Zr_32.2_ (Fig. 5). By comparing the partial density of states for Cu and Zr to the total density of states (Fig. 5a), it can be observed that there is a strong hybridisation at approximately −4 eV. A completely different bonding behaviour can be observed for the Pd_57.0_Al_23.9_Cu_11.4_Y_7.7_ glass depicted in Fig. 5c. With the two main constituents of this glass, Pd and Al, populating different energy levels, the overall degree of hybridisation is low. We thus propose that solids with a small contribution of hybridised bonds to the overall bond character, and therefore less directional bonding and topological connectivity, resist brittle fracture and deform by atomic scale shearing. Hence, it can be inferred that with Pd and Al populating different energy levels, the species are more likely to accommodate shear and facilitate the generation of shear transformation zones^45^. This notion is consistent with the bonding analysis of Pd_57.0_Al_23.9_Cu_11.4_Y_7.7_, as shown in Fig. 6, where antibonding interactions between Pd and Al are observed for energies below −7.5 eV. A similar behaviour is observed for the tough Pt_57.5_Cu_14.7_Ni_5.3_P_22.5_ glass^3^, which shows antibonding interactions within the same energy range. However, in the Au_49_Ag_5.5_Pd_2.3_Cu_26.9_Si_16.3_ glass, Au-Si interactions induce the population of bonding states for all energies below the Fermi energy; this is also consistent with a fracture energy that is an order of magnitude lower^18^ than that of the tough Pt_57.5_Cu_14.7_Ni_5.3_P_22.5_ glass^3^. The bonding states analysis for the Au_49_Ag_5.5_Pd_2.3_Cu_26.9_Si_16.3_ bulk metallic glass from Fig. 6 is consistent with the density of states analysis in Fig. 7. Figure 7a,b present the total and partial density of states for the Au_49_Ag_5.5_Pd_2.3_Cu_26.9_Si_16.3_ and Pt_57.5_Cu_14.7_Ni_5.3_P_22.5_ bulk metallic glass, respectively. From Fig. 7a it can be observed that in the partial density of states for Si, the 3s orbitals are occupied at about −9 eV. Hybridisation is observed between Si, Cu, Pd and Au at these energy levels, which is consistent with the bonding state analysis conducted in Fig. 6, where strong Au-Si bonding interactions can be observed. From Fig. 7b, a low degree of hybridisation especially between P and Pt can be observed for the ductile Pt_57.5_Cu_14.7_Ni_5.3_P_22.5_ bulk metallic glass. This observation is consistent with the density of states analysis for the ductile Pd_57.0_Al_23.9_Cu_11.4_Y_7.7_ thin film metallic glass and the bonding analysis from Fig. 6. Hence, through the electronic structure analysis, we are able to distinguish between order of magnitude changes in the fracture energy: a tough behaviour is observed for Pt_57.5_Cu_14.7_Ni_5.3_P_22.5_^3^, whereas Au_49_Ag_5.5_Pd_2.3_Cu_26.9_Si_16.3_ exhibits reduced toughness with a fracture energy that is an order of magnitude lower^18^, while all brittle systems have a large population of strongly bonded states. Hence, the electronic structure analysis is consistent with the fracture surface analysis and fracture toughness data presented here for the thin film metallic glasses as well as all available literature data on bulk metallic glasses^3^,^12^,^13^,^18^. Furthermore, it is reported in the literature that oxide and chalcogenide glasses exhibit strong hybridisation^14^,^15^,^16^,^17^, which is also consistent with the notion put forward here. Structural heterogeneity, and its importance for mechanical properties of metallic glasses, is widely discussed and under on-going evaluation in the literature^35^,^36^,^37^. From the combined evaluation of the critical defect size and the atom probe tomography analysis, we can exclude chemically induced structural heterogeneities and structural heterogeneities, which exhibit density fluctuations with sizes larger than 2 nm. It is inferred that the thin film metallic glasses are in similar states of low structural relaxation owing to the fast quenching rates employed here^8^. Literature suggests that a low structural relaxation state promotes the formation of shear transformation zones^8^,^35^. For the thin film metallic glasses studied here, we observe large fracture strength and no yielding before fracture except for the Pd-based metallic glass. The presence of a large density of shear transformation zones would suggest an easy onset of shear and, hence, low yield strength, which we, however, do not observe for the thin film metallic glasses studied here. Per our computational approach and the size of the cells utilised, structural heterogeneities exceeding 1.5 nm are not present. Hence, we infer that differences in structural heterogeneity, which may be present in our thin film metallic glass at length scales smaller than 2 nm, do not dominate the chemical composition-induced changes in the electronic structure as well as the associated changes in the mechanical behaviour.

The outcome of the electronic structure comparison is that hybridisation is the fingerprint and hence a suitable predictor of high damage tolerance in thin film metallic glasses, which is consistent with the electronic structure analysis of oxide glasses^14^, chalcogenides^15^,^16^,^17^; Fe-based glasses^12^, Zr-based glasses^11^,^12^, Pt_57.5_Cu_14.7_Ni_5.3_P_22.5_^3^, Au_49_Ag_5.5_Pd_2.3_Cu_26.9_Si_16.3_^18^ bulk glasses and all five thin film systems synthesised and evaluated here. Hence, our data as well as all available literature data on the subject are consistent with the conclusion drawn here.

Additionally, to evaluate the change in the bonding nature upon deformation, the density of states in a sheared state is studied. For the Cu_67.8_Zr_32.2_ thin film metallic glass exposed to 5% shear, the centre of mass for the total density of states shifts by only 0.05 eV compared to the unstrained glass. On the other hand, the total density of states in the Pd_57.0_Al_23.9_Cu_11.4_Y_7.7_ thin film metallic glass exposed to 5% shear shifts by 0.3 eV towards the Fermi level. Hence, the thin film metallic glass becomes softer upon shearing, which explains the onset of plasticity and the high toughness observed.

## Discussion

Based on correlative theoretical and experimental data, we put forward the notion that the fraction of bonds stemming from hybridised states compared to the overall bonding can serve as a measure for damage tolerance, reflecting the electronic nature of the mechanisms involved. A low fraction of hybridised states yields an easy shear relaxation, thus promoting the formation of shear transformation zones, which initiates plastic deformation and bond switching. This notion is supported not only by our data but also by published literature on the electronic structure of Zr-Ni-Al bulk metallic glasses^12^, in which the directional covalent bonding between Ni and Al is shown to induce a decrease in toughness (Fig. 3b). In addition, it has been reported that large plasticity in Fe-based bulk metallic glasses can be achieved when “clusters are linked with metal-metal bonds”, according to Yang *et al*.^13^. This assessment for Fe-based bulk metallic glasses is in line with the conclusions drawn here for thin film metallic glasses. Furthermore, it is reported in the literature that the brittle oxide and chalcogenide glasses exhibit strong hybridisation^14^,^15^,^16^,^17^, which is consistent with the notion presented here. In contrast to the notion of a sharp transition from brittle to ductile behaviour within a Poisson ratio range from 0.31 to 0.32^5^, we provide an electronic fingerprint for damage-tolerant thin film metallic glasses. The notion put forward is consistent with the thin film metallic glass data presented here as well as with all available data from the literature^3^,^11^,^12^,^13^,^14^,^15^,^16^,^17^,^18^. The fraction of bonds stemming from hybridised states compared to the overall bonding is identified as the electronic fingerprint for the outstanding damage-tolerance of the Pd_57.0_Al_23.9_Cu_11.4_Y_7.7_ thin film metallic glass with a strength exceeding 4 GPa and fracture toughness of 49 MPam^1/2^, which is an order of magnitude larger than that of ultrahigh-strength bulk metallic glasses^46^.

Whereas the Poisson ratio alone is not a sufficient criterion to determine the brittle-ductile transition of glasses, it constitutes a valuable alloying guideline and is hence an important first-order material design criterion. By identifying the electronic structure changes associated with the transition from brittle to tough behaviour, this work enables the knowledge-based design of novel damage-tolerant thin film metallic glasses.

## Methods

### Density functional theory*-*based *ab initio* molecular dynamics simulations

To obtain the elastic properties of the glass systems and ensure a realistic representation of the short-range order, the method from Schnabel *et al*.^47^ was applied; this method has previously been validated by Hostert *et al*.^48^. *Ab initio* molecular dynamics simulations were performed with the OpenMX code^49^ based on density functional theory^50^. An N-point grid larger than 72 × 72 × 72 and a cutoff energy of 150 Ry were used. Electronic potentials, with basis functions in the form of a linear combination of localised pseudo-atomic orbitals and a generalised gradient approximation, were used^51^,^52^. As an initial configuration a body centred cubic structure cell was applied. By an ad-hoc approach 115 atoms and 13 vacancies were distributed on the lattice sites^48^. The amorphous structure was obtained through heating each cell to 4000 K by scaling the velocity for 400 fs and subsequent instant quenching to 0 K^48^. The heating and quenching cycle was performed up to four times until the equilibrium volume converged within 2%. For the ground state calculations spin polarization was considered, whereas it was neglected in the simulations of the melt. The elastic properties and density of states were obtained from the stress-free and relaxed configurations at the ground state for which the Vienna Atomic Simulation Package^53^, was applied^48^. The bulk and shear modulus were obtained through the Birch-Murnaghan equation of state^54^, whereas the shear modulus was calculated according to the Hill approximation^55^. The Poisson ratio was obtained through a combination of the bulk and shear modulus^47^. The typical error in the Poisson ratio was evaluated by sampling the Cu_70_Zr_30_ metallic glass five times. For the sampling, an identical initial configuration, and iterative heating and quenching cycle were used. The standard deviation of the Poisson’s ratio obtained was 0.002, which corresponds to an error of 0.6% and is within the size of the symbols in Figs 3 and 4. Furthermore, the densities of states were evaluated for the configurations used for obtaining *C*_*44*_. For the Pt_57.5_Cu_14.7_Ni_5.3_P_22.5_^3^ and Au_49_Ag_5.5_Pd_2.3_Cu_26.9_Si_16.3_ systems^18^ synthesised and tested in the literature, mechanical property reports were completed along with electronic structure information^56^,^57^,^58^.

### Synthesis

Pd_57.0_Al_23.9_Cu_11.4_Y_7.7_, Co_42.0_Fe_13.2_Zr_10.7_Ta_6.6_B_27.5_, Co_58.4_Fe_7.0_B_34.6_, Cu_67.8_Zr_32.2_ and Co_68.3_Zr_31.7_ metallic glass thin films were synthesised by physical vapour deposition^59^ by employing magnetron sputtering from 50 mm targets with a diameter of 50 mm using a ultrahigh-vacuum system^59^. Within this work, we report the synthesis and properties of the Pd_57.0_Al_23.9_Cu_11.4_Y_7.7_ metallic glass thin film for the first time. We have chosen the Pd_57.0_Al_23.9_Cu_11.4_Y_7.7_ thin film metallic glass because it exhibits an identical Poisson’s ratio as the Cu_70_Zr_30_ thin film metallic glass but a different chemical bonding nature. Single-crystal two-inch (001) Si wafers were used as the substrate material. The ultrahigh-vacuum system applied was a lab scale, self-built system equipped with a load lock for sample transfer. The system reached base pressures below 2·10^−6^ Pa and the working pressure applied was 0.4 Pa. Ar with a purity of 99.9999% was used as the sputtering gas. The magnetrons were tilted by 19° normal to the substrate. The substrate-to-target distance was fixed to 10 cm. All thin film metallic glasses were synthesised with a substrate rotation of 30 rounds per minute. Direct current and radio frequency power supplies were utilised for metallic and boron targets, respectively. The target power densities with respect to the whole target area were 5.1, 6.1, 2.0 and 5.1 W/cm^2^ for Pd, Al, Cu and Y, respectively. A target power density of 1.9 W/cm^2^ was used for a Co 88at%/Ta 12at% compound target for the Co-Fe-Zr-Ta-B system, whereas the target power densities for Fe, Zr and B were 0.4, 1.1 and 8.7 W/cm^2^, respectively. Target power densities of 1.9, 0.2 and 8.7 W/cm^2^ were applied for the Co-Fe-B system. Target power densities of 3.1 and 5.3 W/cm^2^ were applied for the Cu-Zr system, and 5.1 and 5.9 W/cm^2^ were utilised for the Co-Zr system.

### Atom probe tomography

To investigate the homogenous chemical distribution of the sputtered samples, we performed three-dimensional atom probe tomography (3D-APT) measurements. 3D-APT samples of all five metallic glass thin films were prepared using a FIB (FEI Helios Nanolab 600i dual-beam FIB) equipped with a micromanipulator. The APT tips were prepared according to a standard lift-out procedure^60^, and final shaping was performed with low energy (5 keV) to prevent Ga-implantation. The APT measurements were performed on a commercial local electrode atom probe (LEAP 3000X HR, CAMECA Instruments) in voltage mode at a base temperature of 60 K, pulse repetition rate of 200 kHz, pulse fraction of 15% and target evaporation rate of 1%. The acquired datasets were analysed using the reconstruction software IVAS 3.6.8^61^ (CAMECA Instruments). In particular, frequency distribution analysis was used to determine the homogeneity of the compositions (with a block size of 100 atoms).

### Cantilever deflection experiments

The Si substrate was etched using 30% KOH solution at 70 °C to obtain free-standing thin films of the sputtered metallic glasses. Focused ion-beam (FEI Helios Nanolab 600i dual-beam FIB) milling was used to prepare the micro-cantilevers. Coarse cuts were made at 2.5 nA (30 kV), and final polishing was performed at 80 pA (30 kV) to obtain cantilevers of approximately 18 μm × 2.5 μm × 2.5 μm. The cantilevers for the fracture toughness measurements were pre-notched at a lower current of 7.7 pA (30 kV). Both the notched and un-notched specimens were loaded *in situ* into the scanning electron microscope (JEOL-JSM 2000) using the ASMEC UNAT-2 indenter. A conical tip (1 μm tip radius) was used to load all microbeams under displacement control at a constant rate of 5 nm/s. The loading sequence involved numerous loads/unloads over multiple cycles. The pop-in/fracture load corresponding to the crack propagation event in the notched beams was used as an input in the analytical formula for determining K_IC_ from either linear elastic fracture mechanics (LEFM) for the brittle glasses or J-integral measurements for the ductile metallic glasses. The testing technique and analysis follows the procedure established by Matoy *et al*.^24^ and Wurster *et al*.^25^ for determining K_IC_ and J_IC_, respectively. The fracture toughness of the Co_68.3_Zr_31.7_ thin films was also calculated using extended finite element simulations of the cantilever bending in Abaqus 10.1 to crosscheck the validity of the results from the analytical formula. The fracture load of the unnotched beams was used to determine the maximum bending (yield/fracture) strength *σ*_*f*_^24^. A total of 80 samples were tested, with 8 un-notched and 8 notched beams for each composition. The error bars for the four metallic glass systems studied here are within the size of the symbol in Fig. 3. The beams were imaged both before and after fracture to follow the crack trajectory.

The strain energy release rate (fracture energy) (G) is obtained from equation (3) 62 using either the K_IC_ or J_IC_ values for all metallic glass thin films:


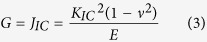


The plane strain condition is fulfilled for


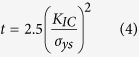


where t represents the relevant specimen dimensions. The plastic zone sizes were calculated from continuum mechanics using the measured K_IC_ and strength data for all the metallic glass thin films (Fig. 2f). We use fracture strength data instead of yield strength data for the brittle metallic glasses because they fractured prior to yielding. Hence, the plastic zone sizes reported here are overestimates of the actual sizes. These show that the cantilever beam dimensions are at least 20 times larger than the plastic zone size at the crack tip except for the ductile Pd-based metallic glass. Furthermore, for metallic glasses in particular, the fracture surface features are supposed to be representative of the plastic zone size^1^. The fracture surface features are an even smaller fraction of the cantilever dimensions, not exceeding 60 nm for the most ductile glass. In the case of the Pd-based glass, we have used fracture toughness results from the J-integral calculations, which could be sample size dependent.

## Additional Information

**How to cite this article**: Schnabel, V. *et al*. Electronic hybridisation implications for the damage-tolerance of thin film metallic glasses. *Sci. Rep.* **6**, 36556; doi: 10.1038/srep36556 (2016).

**Publisher’s note:** Springer Nature remains neutral with regard to jurisdictional claims in published maps and institutional affiliations.

## References

[b1] Schuh C. A., Hufnagel T. C. & Ramamurty U. Mechanical behavior of amorphous alloys. Acta Mater. 55, 4067–4109 (2007).

[b2] Demetriou M. D. . A damage-tolerant glass. Nature Mater. 10, 123–128 (2011). 21217693 10.1038/nmat2930

[b3] Schroers J. & Johnson W. L. Ductile Bulk Metallic Glass. Phys. Rev. Lett. 93, 255506 (2004). 15697909 10.1103/PhysRevLett.93.255506

[b4] Greaves G. N., Greer A. L., Lakes R. S. & Rouxel T. Poisson’s ratio and modern materials. Nature Mater. 10, 827–837 (2011). 10.1038/nmat3134 22020006

[b5] Lewandowski J. J., Wang W. H. & Greer A. L. Intrinsic plasticity of brittleness of metallic glasses. Philos. Mag. Lett. 85, 77–87 (2005).

[b6] Jia P., Zhu Z.-d., Ma E. & Xu J. Notch toughness of Cu-based bulk metallic glasses. Scripta Mater. 61, 137–140 (2009).

[b7] Kim C. P. . Fracture toughness study of new Zr-based Be-bearing bulk metallic glasses. Scripta Mater. 60, 80–83 (2009).

[b8] Kumar G., Neibecker P., Liu Y. H. & Schroers J. Critical fictive temperature for plasticity in metallic glasses. Nature Com. 4, 1536 (2013). 10.1038/ncomms2546 PMC3586724 23443564

[b9] Zhao Y.-Y. . Composition Effect on Intrinsic Plasticity or Brittleness in Metallic Glasses. Sci. Rep. 4, 5733 (2014). 25043428 10.1038/srep05733 PMC4104394

[b10] Zheng N. . Design of ductile bulk metallic glasses by adding “soft” atoms. Appl. Phys. Lett. 100, 141901 (2012).

[b11] Raghavan R., Murali P. & Ramamurty U. On factors influencing the ductile-to-brittle transition in a bulk metallic glass. Acta Mater. 57, 3332–3340 (2009).

[b12] He Q., Cheng Y.-Q., Ma E. & Xu J. Locating bulk metallic glasses with high fracture toughness: Chemical effects and composition optimization. Acta Mater. 59, 202–215 (2011).

[b13] Yang W. . Mechanical properties and structural features of novel Fe-based bulk metallic glasses with unprecedented plasticity. Sci. Rep. 4, 6233 (2014). 25167887 10.1038/srep06233 PMC5385824

[b14] Wu M., Liang Y., Jiang J.-Z. & Tse J. S. Structure and Properties of Dense Silica Glass. Sci. Rep. 2, 398 (2012). 22570763 10.1038/srep00398 PMC3347315

[b15] Deringer V. L. . Bonding Nature of Local Structural Motifs in Amorphous GeTe. Angew. Chem. Int. Ed. 53, 10817–10820 (2014). 10.1002/anie.201404223 25044627

[b16] Guin J.-P., Rouxel T. & Sangleboeuf J.-C. Hardness, Toughness, and Scratchability of Germanium–Selenium Chalcogenide Glasses. J. Am. Ceram. Soc. 85, 1545–1552 (2002).

[b17] O’Reilly E. P. The electronic structure of Ge-Se and Ge-Te compounds. J. Phys. C: Solid State Phys. 15, 1449–1455 (1982).

[b18] Madge S. V., Louzguine-Luzgin D. V., Lewandowski J. J. & Greer A. L. Toughness, extrinsic effects and Poisson’s ratio of bulk metallic glasses. Acta Mater. 60, 4800–4809 (2012).

[b19] Kumar G., Prades-Rodel S., Blatter A. & Schroers J. Unusual brittle behavior of Pd-based bulk metallic glass. Scripta Mater. 65, 585–587 (2011).

[b20] Park E. S. & Kim D. H. Phase separation and enhancement of plasticity in Cu–Zr–Al–Y bulk metallic glasses. Acta Mater. 54, 2597–2604 (2006).

[b21] Barbee T. W., Holmes W. H., Keith D. L. & Pyzyna M. K. Synthesis of Amorphous Niobium-Nickel Alloys by Vapor Quenching. Thin Solid Films 45, 591–599 (1977).

[b22] Moody M. P., Stephenson L. T., Ceguerra A. V. & Ringer S. P. Quantitative Binomial Distribution Analyses of Nanoscale Like-Solute Atom Clustering and Segregation in Atom Probe Tomography Data. Microsc. Res. Tech. 71, 542–550 (2008). 18425800 10.1002/jemt.20582

[b23] Schnabel V. . Revealing the relationships between chemistry, topology and stiffness of ultrastrong Co-based metallic glass thin films: A combinatorial approach. Acta Mater. 107, 213–219 (2016).

[b24] Matoy K. . A comparative micro-cantilever study of the mechanical behavior of silicon based passivation films. Thin Solid Films 518, 247–256 (2009).

[b25] Wurster S., Motz C. & Pippan R. Characterization of the fracture toughness of micro-sized tungsten single crystal notched specimens. Philos. Mag. 92, 1803–1825 (2012).

[b26] Lee S.-W., Huh M.-Y., Fleury E. & Lee J.-C. Crystallization-induced plasticity of Cu–Zr containing bulk amorphous alloys. Acta Mater. 54, 349–355 (2006).

[b27] Malandro D. L. & Lacks D. J. Relationships of shear-induced changes in the potential energy landscape to the mechanical properties of ductile glasses. J. Chem. Phys. 110, 4593–4601 (1999).

[b28] Johnson W. L. & Samwer K. A Universal Criterion for Plastic Yielding of Metallic Glasses with a (T/T_g_)^2/3^ Temperature Dependence. Physical Review Letters 95, 195501 (2005). 16383993 10.1103/PhysRevLett.95.195501

[b29] Fujita K. . Fatigue properties in high strength bulk metallic glasses. Intermetallics 30, 12–18 (2012).

[b30] Jaya B. N., Kirchlechner C. & Dehm G. Can microscale fracture tests provide reliable fracture toughness values? A case study in silicon. J. Mater. Res. 30, 686–698 (2015).

[b31] Jaya B. N. & Jayaram V. Fracture Testing at Small-Length Scales: From Plasticity in Si to Brittleness in Pt. JOM 68, 94–108 (2016).

[b32] Sebastiani M., Johanns K. E., Herbert E. G. & Pharr G. M. Measurement of fracture toughness by nanoindentation methods: Recent advances and future challenges. Curr. Op. Solid State. Mater. Sci. 19, 324–333 (2015).

[b33] Elswijk H. B., Bronsveld P. M. & Hosson J. T. M. D. Field Ion Microscope, Imaging Atom Probe Study of Metallic Glasses. Journal De Physique 11, C6–305 (1987).

[b34] Miller M. K., Longstreth-Spoor L & Kelton K. F Detecting density variations and nanovoids. Ultramicroscopy 111, 469–472 (2011). 21664542 10.1016/j.ultramic.2011.01.027

[b35] Ding J., Patinet S., Falk M. L., Cheng Y. & Ma E. Soft spots and their structural signature in a metallic glass. 111, 14052–14056 (2014). 10.1073/pnas.1412095111 PMC4191783 25228762

[b36] Ketov S. V. . Rejuvenation of metallic glasses by non-affine thermal strain. Nature 524, 200–203 (2015). 26268190 10.1038/nature14674

[b37] Ma E. & Ding J. Tailoring structural inhomogeneities in metallic glasses to enable tensile ductility at room temperature. Materials Today 00, 10.1016/j.mattod.2016.04.001 (2016).

[b38] Ashby M. F. Materials Selection in Mechanical Design Ch. 6, 105–172 (Oxford, 2005).

[b39] Ashby M. F. & Greer A. L. Metallic glasses as structural materials. Scripta Mater. 54, 321–326 (2006).

[b40] Lewandowski J. J., Gu X. J., Nouri A. S., Poon S. J. & Shiflet3 G. J. Tough Fe-based bulk metallic glasses. Appl. Phys. Lett. 92, 091918 (2008).

[b41] Wesseling P., Nieh T. G., Wang W. H. & Lewandowski J. J. Preliminary assessment of flow, notch toughness, and high temperature behavior of Cu_60_Zr_20_Hf_10_Ti_10_ bulk metallic glass. Scripta Mater. 51, 151–154 (2004).

[b42] Xi X. K. . Fracture of Brittle Metallic Glasses: Brittleness or Plasticity. Phys. Rev. Lett. 94, 125510 (2005). 15903937 10.1103/PhysRevLett.94.125510

[b43] Mecholsky J. J., Rice R. W. & Freiman S. W. Prediction of Fracture Energy and Flaw Size in Glasses from Measurements of Mirror Size. J. Am. Ceram. Soc. 57, 440–443 (1974).

[b44] Nollmann N., Binkowski I., Schmidt V., Rösner H. & Wilde G. Impact of micro-alloying on the plasticity of Pd-based bulk metallic glasses. Scripta Mater. 111, 119–122 (2016).

[b45] Greer A. L., Cheng Y. Q. & Ma E. Shear bands in metallic glasses. Mater. Sci. Eng., R 74, 71–132 (2013).

[b46] Inoue A., Shen B. L., Koshiba H., Kato H. & Yavari A. R. Ultra-high stength above 5000 MPa and soft magnetic properties of Co-Fe-Ta-B bulk glassy alloys. Acta Mater. 52, 1631–1637 (2004).

[b47] Schnabel V., Evertz S., Rueß H., Music D. & Schneider J. M. Stiffness and toughness prediction of Co-Fe-Ta-B metallic glasses, alloyed with Y, Zr, Nb, Mo, Hf, W, C, N and O by *ab initio* molecular dynamics. J. Phys.: Condens. Matter 27, 105502 (2015). 25710383 10.1088/0953-8984/27/10/105502

[b48] Hostert C. . *Ab initio* molecular dynamics model for density, elastic properties and short range order of Co-Fe-Ta-B metallic glass thin films. J. Phys.: Condens. Matter 23, 475401 (2011). 22056956 10.1088/0953-8984/23/47/475401

[b49] Ozaki T. & Kino H. Efficient projector expansion for the ab initio LCAO method. Phys. Rev. B 72, 045121 (2005).

[b50] Hohenberg P. & Kohn W. Inhomogeneous Electron Gas. Phys. Rev. 136, 864–871 (1964).

[b51] Sholl D. S. Density functional theory: a practical introduction. (John Wiley & Sons, Inc., 2009).

[b52] Ozaki T. Variationally optimized atomic orbitals for large-scale electronic structures. Phys. Rev. B 67, 155108 (2003).

[b53] Kresse G. & Fürthmüller J. Efficient iterative schemes for *ab initio* total-energy calculations using a plane-wave basis set. Phys. Rev. B 54, 11169 (1996). 10.1103/physrevb.54.11169 9984901

[b54] Birch F. Finite Strain Isotherm and Velocities for Single-Crystal and Polycrystalline NaCl at High Pressures and 300°K. J. Geoph. Res. 83 (1978).

[b55] Holm B., Ahuja R., Yourdshadyan Y., Johansson B. & Lundqvist B. I. Elastic and optical properties of a- and k-Al_2_O_3_. Phys. Rev. B 59, 12777 (1999).

[b56] Dronskowski R. & Blöchl P. E. Crystal Orbital Hamilton Populations (COHP). Energy-Resolved Visualization of Chemical Bonding in Solids based on Density-Functional Calculations. J. Phys. Chem. 97, 8617–8624 (1993).

[b57] Deringer V. L., Tchougreeff A. L. & Dronskowski R. Crystal Orbital Hamilton Population (COHP) Analysis as Projected from Plane-Wave Basis Sets. J. Phys. Chem. A 115, 5461–5466 (2011). 21548594 10.1021/jp202489s

[b58] Maintz S., Deringer V. L., Tchougreeff A. L. & Dronskowski R. Analytic Projection from Plane-Wave and PAW Wavefunctions and Application to Chemical-Bonding Analysis in Solids. J. Comput. Chem. 34, 2557–2567 (2013). 24022911 10.1002/jcc.23424

[b59] Schnabel V. . Temperature-Induced Short-Range Order Changes in Co67B33 Glassy Thin Films and Elastic Limit Implications. Mater. Res. Lett. 3, 82–87 (2015).

[b60] Thompson K. . *In situ* site-specific specimen preparation for atom probe tomography. Ultramicroscopy 107, 131–139 (2007). 16938398 10.1016/j.ultramic.2006.06.008

[b61] Larson D. J., Prosa T. J., Ulfig R. M., Geiser B. P. & Kelly T. F. Local Electrode Atom Probe Tomography. (Springer, New York Heidelberg Dordrecht London, 2013).

[b62] ASTM E 1820-01, *Standard test method for measurement of fracture toughness.* http://dx.doi.org/10.1520/e1820-01 (2001) (Date of access: 03/10/2016).

